# Professional Ethics

**Published:** 1891-01

**Authors:** S. B. Bartholomew

**Affiliations:** Springfield, Mass.


### PROFESSIONAL ETHICS.[1]

BY DR. S. B. BARTHOLOMEW, SPRINGFIELD, MASS.

PROFESSIONAL ethics is a system of morals formulated out of unwritten laws that by common consent are recognized as best in associated life.

[1] Read at the Union Dental Meeting, Boston, October 28, 1890.

All systems of ethics recognize the fact that character is developed by that which it feeds on, towards the good or the bad, as the food may be, sometimes by slow, yet sure, gradations.

Nature is never abrupt or impulsive in the development of her creations. The process is sufficiently slow for the survival of the fittest and the attainment of the best, and as the great procession moves along, each man is seen and more nearly justly measured than is generally supposed.

Photography is the art of all arts; truthful in its portraiture of that which is pleasing and repulsive, true or false. It is not limited in its surface views, but penetrates deeper and reveals the forces, and how they act. Nature is the great flash photographer and none can escape the Kodak.

There is a mysterious photographic power that hovers about the atmosphere of a man and pictures, in spite of himself, the power that moves within him. He may spurn all moral codes of ethics, all the science of moral philosophy that teaches men their duty and the reasons for it, all systems of rules for regulating the actions and manners of men in society or associated life, yet his ways, his manners, his peculiarities, and habits of life will conform to as cast-iron a law as was ever formulated, and he will practise the unwritten code so systematically that the name and power behind that code can be read or seen of all men as easily as the scientist can with his microscope show you the microbes of a pestilence.

Not every man in associated life has an ethical standard of what he should be; neither does he realize that every right act goes to the formation of a perfect character, as every wrong act mars or enfeebles it. Men who are most prone to go astray are those without an ideal; or if they have one, there is lacking a persistent endeavor to mould themselves to it. Attainment towards an ideal presupposes a law of habit, and that law must have behind it a persistent moral force. If not, it becomes a law unto itself, and so far as reliable character is concerned, principles of moral action do not enter into it. The way is strewn with men who have fallen out of the race in life just because of this moral ethical defect, and when a man says he does not believe in a code of ethics and will not be bound by it, he does not know whereof he speaks, or else he is bound to be a moral wreck, and if you watch long enough for him you will find him on some one of the highways that lead from whatever profession or calling he may have belonged to, a bad man, on the line that will surely take him, if he pursues it, to Canada.

Statute law protects the most necessary and greater rights that exist among men, but there are other rights that statute law does not reach, that have come down to us through the generations, based on the common rights inherent in social necessities; so that when a man sets up between himself and his neighbor the criminal and statute law and asks himself what does the law allow, he will be a constant trespasser on the moral and ethical rights of his fellow-man.

A code of moral ethics has obtained in associated compacts, and indeed among all classes of people above the criminal. The instinct of this code is so fine and subtle that if a man has any moral sense or conscience prompting him to right actions it will point the way, so that at least his motives will be right though sometimes his actions may be wrong through defective judgment. This code has obtained through the usages of time a supplementary influence over men that is more exacting and controlling than statutes, because it is based on inherent rights, and the man who can, when it suits him, throw overboard this natural and common law, is only a guerilla in the warfare of life, and he is looked upon by his fellows with doubt, mistrust, and often contempt, yes, and is branded as with the mark of Cain, for he has in him all the material that would decide him, if he chose, to cast aside the laws of state or nation, in fact, make himself a rebel against all laws, human or divine.

On us, as a profession, the ethical law of the universe has laid a heavy hand, so that as we work we cannot forget the grandeur and the possible attainments that lie along the path of the true man in his ascent towards the infinite.

A chain is no stronger than its weakest link. A dentist can rise and remain no higher in his professional life than the weakest link in his moral chain can lift and hold him. I am amazed sometimes at the almost total depravity manifested by some of my professional brethren in the way they can go back on their Alma Mater, after she has poured out upon them the wealth of her storehouse, and covered them with the *ægis* of her parchment, proclaiming them professional gentlemen, qualified to do all that may be required of them in the community where they may be located; forgetting all the pledges made to her as sacred as solemn oaths, throwing their code of ethics to the moles and bats; stabbing at every step of the way the profession they have sworn to defend, upbuild, and honor. I have seen the picture of a couple who sat with bowed heads, every line in their faces a furrow of grief that told of hopes blasted through children gone wrong. I said to myself, they were not worth the grief. Instantly over the picture I seemed to see written, "Blood is thicker than water." Look at it as we may, the disgraceful actions of a child reflect only dishonor, the sting of which is mortification and sorrow. This class of men are open in their defiance of the amenities of professional life. We all know them; some of them for a time will be quite strongly intrenched on the public under the cry of professional persecution; but the public soon learn to know them, and they go to their own among the swine. I do not expect by what I may say in this paper to reclaim these sons of perdition, but perhaps some one who may be impatient and almost discouraged over the slow appreciation of merit by the public may be encouraged to wait awhile, and not throw away his manhood for a mess of pottage.

It isn't a bad idea for a man, however well fixed he may be, to sit down for an hour once in a while and examine himself honestly as to his methods and practices in professional life. If you have never done it, do it; you will be surprised at the estimate you will form of yourself, and still more surprised, later on, how near your own estimate, if it is an honest one, will be confirmed by the public and the profession. It is a man's defects that drop him out of the race, and these defects exist because moral ethics do not sufficiently influence his mental forces. Are these mental forces of ours moving along on a sound and healthy plane, looking towards the things that make for a higher manhood? On what plane does the photograph reveal this mental work of ours? Our thoughts are not always spoken in words, but the composite thought is interpreted and stamped on our faces, is read in our walk, in our manners, in our habits, so that our mentality either attracts or repels. Whatever impression our mentality makes on a patient goes with that patient beyond the door of the office, an impelling or drawing force.

The highly trained moral sense of the brain quickens the sense of sight, smell, and love of æsthetic surroundings. A lady enters your office for the first time, a stranger to you personally; instantly the instincts of cultivated tastes put you on trial, and you stand or fall by that judgment she may form of you from your personal appearance and office surroundings. The judgment of this patient will be the judgment of a very large proportion of those patients that you would like to retain. It is very obvious, then, that our mentality should be clean and healthy, pervading the room, the atmosphere, the patient who for the time being occupies the chair, as the perfumes do the flower garden. Such a mentality, trained and disciplined, is an absolute necessity to the highest and most successful business career; it carries with it good judgment and skill and enthusiasm to execute judgment.

Let us look a little further and see if we are quite up to the proper appreciation of a clean standard of morally practical ethics in professional courtesy, those little things done so deftly that the animus may be hidden for a time from the eye and sense of our patients and the public. Whenever the name of a professional brother is a subject of conversation between us and our patient, do we allow professional jealousy to thrust its sting into our remarks? Do we forget the golden rule, "Do unto others as you would that they should do unto you"? Are we in the habit of circulating everything we hear that can possibly injure him? If so, what better are we than filchers of a good name, assassins of character, or petty thieves before a police justice?

> "Who steals my purse, steals trash, 'tis something, nothing;
> 'Twas mine, 'tis his, and has been slave to thousands;
> But he that filches from me my good name,
> Robs me of that which not enriches him,
> And makes me poor indeed."

If what I have just spoken of can stand in an indictment, be assured that it will color and give tone to the atmosphere about us and enter as weights against us in the general estimate of our characters by the public, as well as by the profession.

The moral defects in a man's character can be read in his unspoken thoughts, in his methods in operative and mechanical dentistry. Watch yourself only for a day in thought and act, and see how nearly you will break the spirit, if not the letter, of every commandment in the decalogue. Place all the defects you detect in the one day's operations side by side, and the mental reasoning that accompanies each, and then study that day's history in the light of any code of moral ethics. I fear some of us, at least, would see the handwriting that appeared on the wall at Belteshazzar's feast. One illustration in operative dentistry will cover the whole ground and show how near we come to being criminals. There is an expression common to almost every operator, "There! I guess that will do." It means more with some than others. Let us examine the preparation we have made of a cavity for filling, not one especially prepared for examination, but an average one, when we have laid down the excavator, set aside the engine, and said to ourselves, "I guess that will do." How many of us would be willing to have that examination made by another operator? So in the preparation of the gold and in the filling of the cavity. Do we say, "I guess that will do," when we know it will not do, when we know that the cavity was not properly prepared, and that there are weak spots in the filling that will be fatal to a successful operation? Do we say to ourselves, "That is not right, and it shall be as near right before it is pronounced finished as it is possible for us to make it," or do we give our patient the impression that it is right, and accept the full fee? The merchant who will take advantage of the ignorance of his patrons and sell a piece of cotton-backed for silk velvet is called a liar and a thief by the public, and the law proclaims him a swindler and compels restitution. Our patients are powerless in our hands, hence the necessity of a code of ethics that should be stamped on the moral nature of our profession. The man who examines himself on this line, and acts upon the result of his examination, is the man who grows on the line of the ideal operator, the ideal, conscientious man; his whole life, public as well as professional, becomes tinged by it.

The dentist should be among the foremost men outside of his professional life. With the expiration of his office hours he should be able to turn the key on all professional cares, if his life and business habits are well regulated. What has he to do with his abundant leisure? Give it to recreation and amusement? Its proper share and no more. I say to you, gentlemen, there is no knowledge in the wide fields of science, art, literature, or government that it is not the privilege of the humblest man among us to unlock the secret of each and appropriate their treasures to his own use. A little time well directed each day in active outside pursuits and studies will absorb his restlessness, direct his ambitions, and turn his eyes forward and upward. Work for bread and butter makes of him a simple grad-grind and keeps him on the lower plane, but work as a pleasure, as a duty, as a stepping-stone to higher things, makes of him a full and broad man, sanctifies his ambitions to the benefit of himself and those about him.

The thoughts I have given you in this paper have been culled from a multitude that have been suggested from a close study of the code of ethics under which we, as dentists, are associated. Let me appeal to every young man that has come and is coming into the profession, to make himself familiar with the code that governs the moral action of the men in whose company he has chosen to make the pilgrimage of life, for, be assured, it will give tone and color to the atmosphere about you as you act upon its suggestions or repudiate them. Whichever way you decide to act, a photographic composite picture of your inner self will stand out clear and distinct, so clear and distinct that your professional brethren and the public will form a just estimate of your character and work.

Place then the banner of manhood ethics high in the forefront of your professional and public life, and do not forget that, while you are building to-day, the composite photograph of yourself, your own personal identity, will be stamped on your posterity and tell of your walk and work in life generations after you are gone.

[1] Read before the Massachusetts Dental Society, December, 1889.

